# Cardiac Infiltration in Mycosis Fungoides Resulting in Recurrent Tamponade

**DOI:** 10.1016/j.jaccas.2025.106135

**Published:** 2025-11-19

**Authors:** Ryan A. Karlsson, Conal Houstoun, Micheal Brennan, Ross T. Murphy, Elisabeth Vandenberghe, James Nolan

**Affiliations:** a Department of Cardiology, St James's Hospital, Dublin, Ireland; b Department of Haematology, St James's Hospital, Dublin, Ireland

**Keywords:** cardiac tamponade, cutaneous T-cell lymphoma, mycosis fungoides, pericardial effusion

## Abstract

**Background:**

Clinically apparent cardiac involvement in mycosis fungoides (MF) is a rare phenomenon.

**Case Summary:**

A 61-year-old man presented with dyspnea 11 months after allogeneic hematopoietic stem cell transplantation for MF. Echocardiography revealed a large pericardial effusion causing cardiac tamponade. Emergency pericardiocentesis and effusion analysis confirmed disease relapse isolated to the pericardium in the setting of full donor chimerism. Despite reduction in immunosuppression, relapse with cardiac tamponade later recurred. Combined-modality chemotherapy and radiotherapy was administered. Systemic disease relapse occurred 22 months after isolated pericardial relapse.

**Discussion:**

This unique case of life-threatening isolated cardiac involvement in relapsed MF exposes the heart as a potential sanctuary site that may sequester lymphoma cells from systemic therapies and the graft-versus-lymphoma effect. Optimal management of this rare complication is unknown.

**Take-Home Message:**

Pericardial or myocardial infiltration should be considered in patients with MF presenting with cardiac symptoms, conduction disturbance, or shock.

## Graphical Abstract

**Figure:**
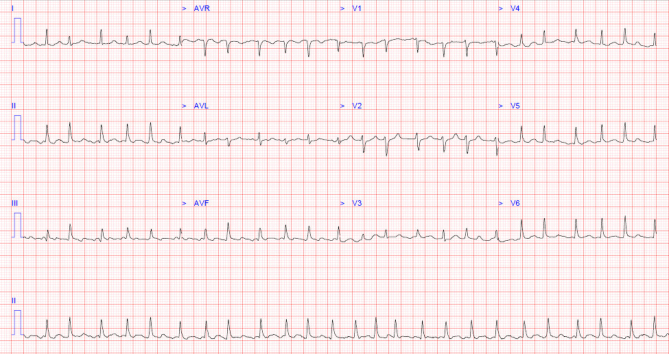

**Visual Summary:**
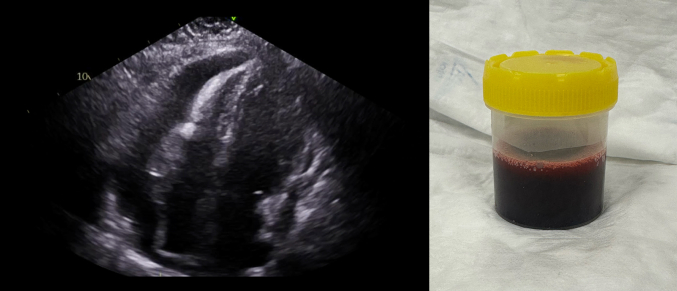
Hemorrhagic Pericardial Effusion Causing Tamponade

## History of Presentation

A 61-year-old man presented to the emergency department 11 months after allogeneic hematopoietic stem cell transplantation (HSCT) for mycosis fungoides (MF), reporting 3 days of acute and progressive dyspnea. On examination, he was hypotensive and tachycardic with cold peripheries and a new oxygen requirement.

**Take-Home Messages**

• Pericardial or myocardial infiltration should be considered in a patient with mycosis fungoides presenting with cardiac symptoms, conduction disturbance, or shock.
• Cardiac involvement in mycosis fungoides may represent a sanctuary site for lymphoma cells against antineoplastic therapies and the graft-versus-lymphoma effect.

## Past Medical History

The patient was diagnosed with erythrodermic MF, a subtype of cutaneous T-cell lymphoma (CTCL), 2 years previously. He received induction chemotherapy with gemcitabine and after a satisfactory response proceeded to matched unrelated donor allogeneic peripheral blood HSCT, receiving reduced-intensity conditioning comprising total skin electron beam therapy, total lymphoid irradiation and antithymocyte globulin,^1^ with ciclosporin and mycophenolate mofetil as graft-versus-host disease (GVHD) prophylaxis. The post-transplant course was complicated by the development of stage 2 steroid-sensitive acute cutaneous GVHD and stage 1 steroid-refractory upper and lower gastrointestinal GVHD, which improved with ruxolitinib. Total and CD3 peripheral blood chimerism were 97% and 95%, respectively, at 100 days, and were 100% from 6 months onward, with negative peripheral blood immunophenotyping for Sézary cells and T-cell clonality by polymerase chain reaction.

Three months after HSCT and 8 months before the onset of events described in this report, the patient was hospitalized owing to acute decompensated heart failure thought secondary to viral (pathogen not identified) myocarditis. Cardiac magnetic resonance imaging at that time had showed concentric left ventricular hypertrophy, diastolic dysfunction, and myocardial edema without fibrosis, infiltration, or infarction. Coronary computed tomography (CT) angiography described minor nonobstructive atheroma. Additional comorbidities included hypothyroidism and benign prostatic hypertrophy. The patient was a nonsmoker and consumed no alcohol.

## Differential Diagnosis

The differential diagnoses for the patient's presentation include septic shock, cardiogenic shock, obstructive shock, unstable tachyarrhythmia, and cardiac GVHD.

## Investigations

**Figure 1:**
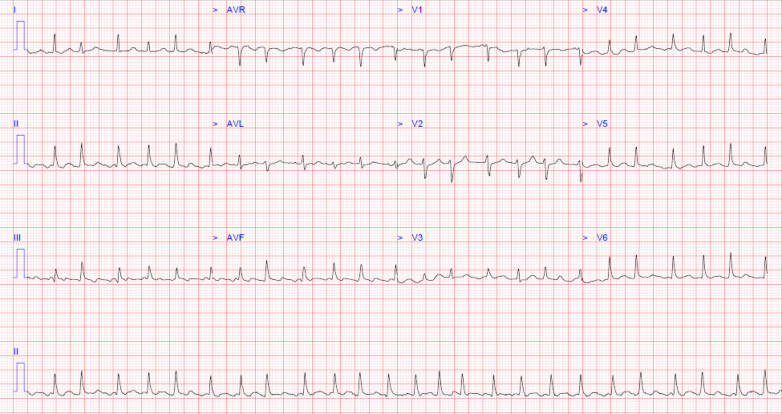
12-Lead ECG 12-lead electrocardiogram showing atrial fibrillation with a rapid ventricular response occurring at a rate of approximately 150 beats/min.

**Figure 2:**
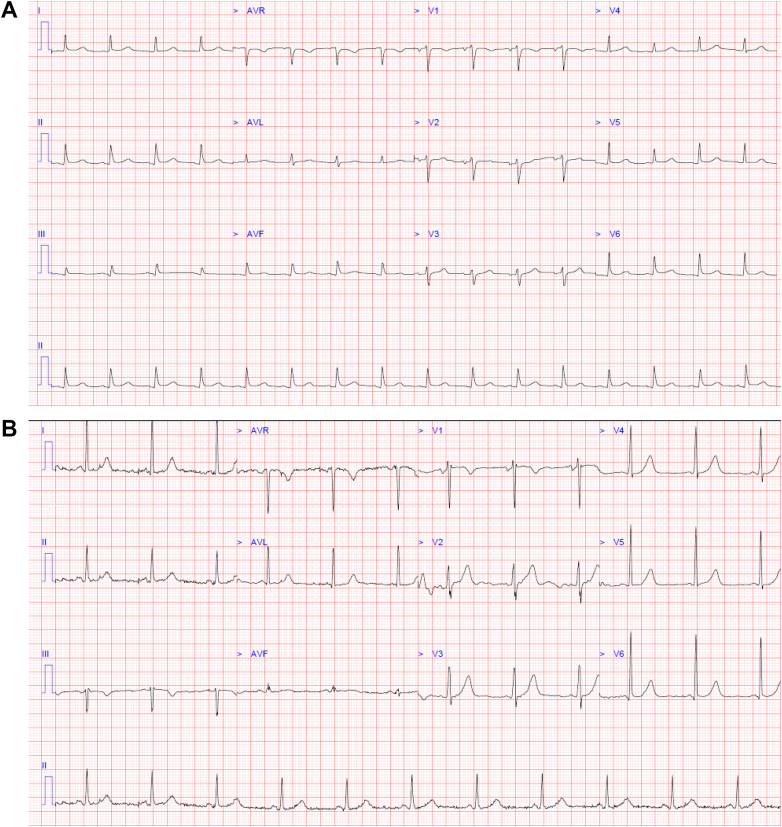
12-Lead ECG (A) 12-lead electrocardiogram showing sinus rhythm with newly developed low-voltage QRS complexes and electrical alternans compared with (B) baseline electrocardiogram.

**Figure 3:**
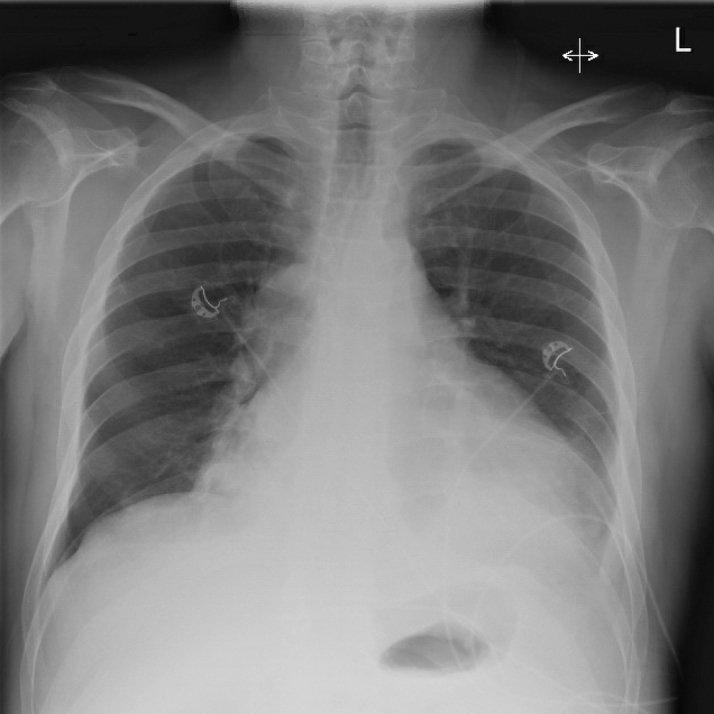
Chest X-Ray Showing an Increased Cardiac Silhouette With a Globular-Shaped Heart

A 12-lead electrocardiogram (ECG) showed atrial fibrillation with a rapid ventricular response (Figure 1), low precordial voltages, and electrical alternans (Figure 2). Blood tests revealed lactic acidosis (pH: 7.26), acute kidney injury, and high-sensitivity troponin T level of 86 ng/L (reference: <14 ng/L). Chest x-ray revealed an enlarged, globular cardiac silhouette (Figure 3). Urgent bedside transthoracic echocardiography (TTE) demonstrated a large global pericardial effusion measuring up to 2.6 cm at the right ventricle, with evidence of right atrial diastolic collapse, as well as a fixed and dilated inferior vena cava, consistent with cardiac tamponade (Video 1, Video 2, Video 3, Video 4, Video 5).

## Management

Emergency pericardiocentesis was performed, draining over 2 L of sanguinous fluid, its appearance concerning for a malignant cause. Molecular analysis of pericardial fluid revealed the presence of clonal T cells with an identical clonal pattern to that of the original cutaneous MF, confirming relapse. Restaging bone marrow aspirate and trephine biopsy alongside CT imaging of the thorax, abdomen, and pelvis did not demonstrate disease elsewhere. Immunosuppressive therapy was discontinued with the aim of inducing a graft-versus-lymphoma effect. Notably, total and CD3 peripheral blood maintained 100% donor chimerism. Cessation of immunosuppression resulted in chronic sclerodermatous cutaneous GVHD, managed initially with recommencement of prednisone and ruxolitinib and ultimately requiring extracorporeal photopheresis. At regular follow-up visits, the patient reported good exercise tolerance. Serial TTE showed preserved left ventricular function without pericardial effusion reaccumulation.

## Outcome and Follow-Up

**Figure 4:**
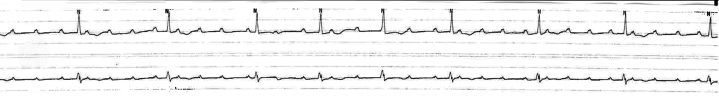
Telemetry Rhythm Strip Displaying Third-Degree AV Block With a Junctional Escape Rhythm Complete AV dissociation can be seen. AV = atrioventricular.

**Figure 5:**
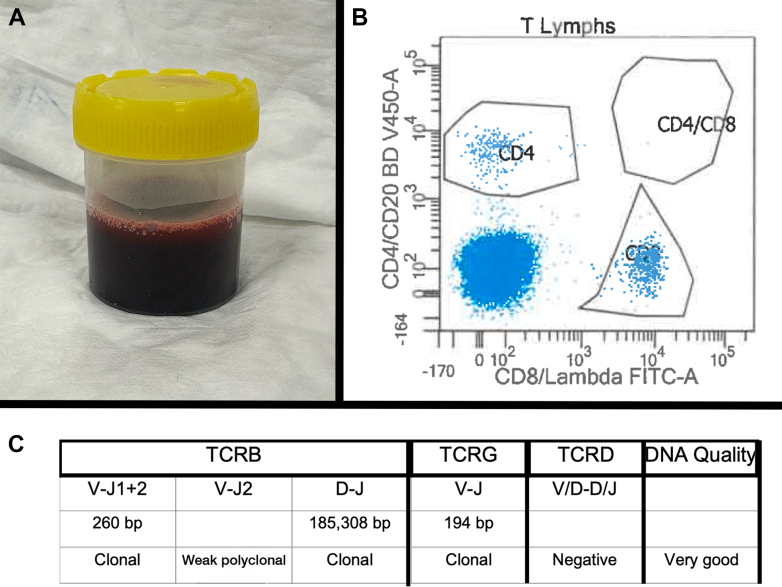
Results of Pericardial Fluid Analysis (A) Hemorrhagic pericardial effusion, with (B) flow cytometry analysis chart revealing a large cluster of CD4-negative and CD8-negative T-lymphocytes, and (C) molecular genetic analysis report confirming the presence of clonal T-cell receptor beta and gamma chain gene rearrangements. These cells matched the profile of monoclonal cells detected within prior samples from the patient's skin and bone marrow in addition to those from the pericardial effusion causing the first occurrence of cardiac tamponade.

At 17 months after pericardiocentesis and just 4 weeks after normal surveillance TTE, the patient re-presented to our hospital with recurrence of a large global pericardial effusion causing cardiac tamponade. Telemetry on admission revealed the development of complete heart block with a junctional escape rhythm (Figure 4). Emergency pericardiocentesis produced approximately 1 L of hemorrhagic fluid, after which atrioventricular conduction improved to intermittent first-degree and Mobitz-1 heart block. Pericardial fluid analysis revealed the presence of clonal T cells once again, confirming that this was a complication of disease relapse (Figure 5).

**Figure 6:**
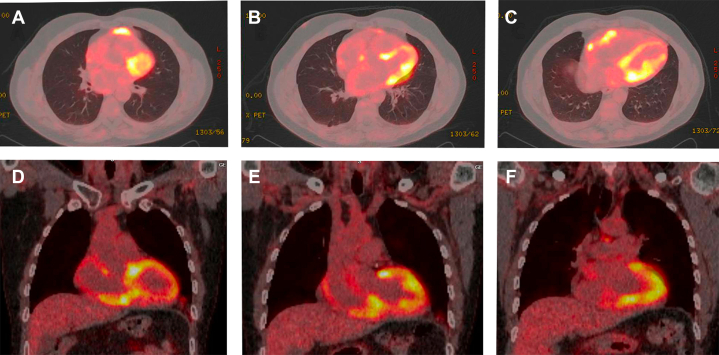
Findings on ^18^F-FDG PET-CT ^18^F-FDG PET-CT (A to C) axial and (D to F) coronal planes revealing intensely FDG-avid fat stranding within the anterior mediastinum directly adjacent to the anterior pericardium (maximum standardized uptake value: 11) and separate FDG-avid fat stranding at the cardiac apex (maximum standardized uptake value: 5), consistent with focal pericardial and overlying mediastinal fat lymphomatous involvement. There is further intense FDG uptake in the region of both ventricles, in particular inferiorly (maximum standardized uptake value: 11.3), in an atypical distribution consistent with pericardial and myocardial involvement rather than physiologic metabolic activity alone. FDG = fluorodeoxyglucose; PET-CT = positron emission tomography–computed tomography.

^18^F-fluorodeoxyglucose (FDG) positron emission tomography (PET)–CT revealed multifocal FDG-avid pericardial and myocardial involvement, a residual small pericardial effusion, and epicardial fat stranding anteriorly and at the cardiac apex, all likely relating to lymphoma deposits (Figure 6). After multidisciplinary discussion, the patient received combined modality therapy with systemic gemcitabine and involved-site cardiac radiotherapy (15 Gy in 5 fractions). At the multidisciplinary heart team meeting, a decision was made not to proceed to permanent pacemaker implantation.

Several months later while awaiting a prophylactic pericardial window procedure, the patient re-presented with exertional dyspnea. TTE revealed a moderate pericardial effusion without tamponade. Repeat CT and ^18^F-FDG PET-CT revealed a 10-cm conglomerate nodal mass encasing the trachea and right and left main bronchi, infradiaphragmatic nodal disease, and extranodal muscular and peritoneal deposits. After thoracoscopic pericardial window formation, material obtained by endobronchial ultrasound again confirmed the presence of clonally identical CD4 and CD8 double-negative T cells, representing relapsed disease with aggressive large cell transformation. The patient went on to receive systemic therapy with gemcitabine and oxaliplatin. Total and CD3 donor chimerism remained 100%.

## Discussion

Mycosis fungoides is a neoplasm characterized by clonal proliferation of small to medium-sized T lymphocytes and is the most common subtype of CTCL, accounting for almost half of all primary cutaneous lymphomas. The typical manifestation of MF involves formation of erythematous patches and infiltrated plaques, later progressing to cutaneous tumors.^2^ Cardiac involvement in MF has been described at autopsy in up to 38% of patients, however clinical manifestations of this are rare, making antemortem diagnosis challenging.^2^ We describe a case of recurrent cardiac tamponade as an isolated site of relapsed MF in a patient with no skin manifestations, with focal pericardial/cardiac relapse preceding systemic relapse by 22 months.

Development of malignancy-related pericardial effusion is caused by direct invasion, lymphatic dissemination, or hematogenous spread of neoplastic cells, which leads to disruption of blood flow or infiltration of the wall of pericardial capillary and small veins, resulting in accumulation of transudate or blood within the pericardial space. A hemorrhagic appearance to pericardial fluid is particularly suggestive of a malignant cause.^3^ Subsequent development of cardiac tamponade depends on both the size of the effusion as well as its rate of accumulation.^3^ In the current case, we describe the development of cardiac tamponade on 2 occasions, including one within 3 months of a normal TTE study, and another within just 4 weeks of an unremarkable surveillance study.

Initial management of patients with cardiac tamponade should include emergency pericardiocentesis, primarily for its therapeutic effect but also to facilitate fluid analysis and establish the etiology of the effusion.^3^ Furthermore, it has been shown that extended pericardial drainage (25 mL or more every 24 hours via intermittent aspiration) is associated with reduced recurrence of cardiac tamponade after initial pericardiocentesis, regardless of the etiology.^4^ In cases of recurrent malignant effusion or tamponade, creation of a pericardial window, typically into the pleural space using video-assisted thoracoscopy or percutaneous balloon pericardiotomy, is indicated to prevent reaccumulation.^5^

Although not understood to be an immune-privileged niche, the pericardial cellular microenvironment is known to be unique and T-cell rich.^6^ The presence of cardiac and pericardial involvement arising in the absence of disease elsewhere is suggestive that this niche could function as a sanctuary site for lymphoma cells against antineoplastic therapies and the graft-versus-lymphoma effect. This phenomenon has rarely been described in hematological malignancies. In our case, the patient's clinical course during episodes of disease relapse was also complicated by the development of both atrial fibrillation and conduction disturbance including complete heart block, which have rarely been described in the setting of CTCL progression and should raise concern for myocardial involvement.^7^

Management options for relapsed MF after allogeneic HSCT include reduction of background immunosuppression, infusion of donor lymphocytes (in the setting of incomplete donor chimerism), and use of systemic chemotherapy or immunomodulatory therapy to which the patient's disease has not previously been refractory. The choice of therapy should also consider the relative risk of inducing acute and chronic GVHD, complications associated with increased morbidity and mortality. Extracorporeal photopheresis and single-agent chemotherapy in the form of gemcitabine or doxorubicin convey a relatively lower GVHD risk.^8^ Treatment of localized recurrence should additionally consider disease site (cutaneous or noncutaneous) and the anticipated toxicity of radiation therapy. Targeted radiotherapy to the heart has demonstrated considerable efficacy in the control of pericardial effusions and reversal of arrythmia in patients with pericardial and myocardial involvement of lymphoma and leukemia, neoplasms which are typically radiosensitive.^9^

To the best of our knowledge, only 2 other cases of cardiac tamponade secondary to progression of MF have been previously described in the literature. Both prior cases describe the development of a malignant pericardial effusion and subsequent cardiac tamponade in the presence of active skin manifestation of the disease and, in one case, other active visceral involvement. In the latter reported case, pericardial-directed radiotherapy was also commenced to prevent effusion reaccumulation after pericardiocentesis, however the patient developed complete heart block and sudden cardiac arrest shortly thereafter.^10^ Our case is unique in that it describes the life-threatening cardiac involvement of MF as the manifestation of relapse in the absence of any other active disease focus, predating systemic relapse by almost 2 years.

## Conclusions

This case serves as a reminder to consider cardiac infiltration and have a lower threshold for cardiac imaging in patients with MF presenting with cardiac symptoms, and it should serve to inform post-treatment surveillance in this patient cohort. The occurrence of clinically significant cardiac involvement of MF demonstrates a novel role for the heart and pericardial niche as a sanctuary site against antineoplastic therapies including the graft-versus-lymphoma effect. A potential mechanism for CTCL immune evasion within the pericardium has not yet been described.

## Funding Support and Author Disclosures

The authors have reported that they have no relationships relevant to the contents of this paper to disclose.

## Appendix

Video 1 Transthoracic Echocardiography Apical 4-Chamber View

Video 2 Transthoracic Echocardiography Apical 4-Chamber View, Right Heart–Focused

Video 3 Transthoracic Echocardiography Parasternal Short-Axis View

Video 4 Transthoracic Echocardiography Parasternal Long-Axis View

Video 5 Transthoracic Echocardiography Subcostal Inferior Vena Cava View
